# Establishment of automated culture system for murine induced pluripotent stem cells

**DOI:** 10.1186/1472-6750-12-81

**Published:** 2012-11-05

**Authors:** Hiroyuki Koike, Koji Kubota, Keisuke Sekine, Takanori Takebe, Rie Ouchi, Yun-Wen Zheng, Yasuharu Ueno, Naoki Tanigawa, Hideki Taniguchi

**Affiliations:** 1 Department of Regenerative Medicine, Graduate School of Medicine, Yokohama City University, 3-9 Fukuura, Kanazawa-ku, Yokohama, Kanagawa 236-0004, Japan; 2 Chiyoda Corporation, Minatomirai Grand Central Tower, 4-6-2 Minatomirai, Nishi-ku, Yokohama, Kanagawa 220-8765, Japan

**Keywords:** Induced pluripotent stem (iPS) cell, Automated cell culture system (ACCS), CO_2_ incubator-scale, Pluripotency

## Abstract

**Background:**

Induced pluripotent stem (iPS) cells can differentiate into any cell type, which makes them an attractive resource in fields such as regenerative medicine, drug screening, or *in vitro* toxicology. The most important prerequisite for these industrial applications is stable supply and uniform quality of iPS cells. Variation in quality largely results from differences in handling skills between operators in laboratories. To minimize these differences, establishment of an automated iPS cell culture system is necessary.

**Results:**

We developed a standardized mouse iPS cell maintenance culture, using an automated cell culture system housed in a CO_2_ incubator commonly used in many laboratories. The iPS cells propagated in a chamber uniquely designed for automated culture and showed specific colony morphology, as for manual culture. A cell detachment device in the system passaged iPS cells automatically by dispersing colonies to single cells. In addition, iPS cells were passaged without any change in colony morphology or expression of undifferentiated stem cell markers during the 4 weeks of automated culture.

**Conclusions:**

Our results show that use of this compact, automated cell culture system facilitates stable iPS cell culture without obvious effects on iPS cell pluripotency or colony-forming ability. The feasibility of iPS cell culture automation may greatly facilitate the use of this versatile cell source for a variety of biomedical applications.

## Background

Since the development of induced pluripotent stem (iPS) cells, their use has been anticipated in various areas, including regenerative medicine and drug discovery [1,2]. The advantages of iPS cells include their multipotency, and they can be established from individuals, allowing the creation of pluripotent stem cells from any donor with any genetic background [3-5]. Hepatocytes derived from iPS cells are useful in evaluating drug sensitivity and toxicity and also in understanding highly variable pathological conditions [6,7]. Obtaining mature cells like hepatocytes for drug development is impeded by short supply, high cost, and variable quality [8]. To solve these problems, directed differentiation of iPS cells into somatic lineages *in vitro* has been attempted extensively [9-11]. Once the creation of mature, functional hepatocytes from iPS cells is successful, the development of stable supply system of iPS cells will be necessary for their translation to these applications.

In this regard, it is important to establish an automated cell culture system (ACCS), which facilitates stable and standardized iPS cell culture and enables researchers to handle sufficient quantities of iPS cells. To date, such ACCS are difficult to handle in a space-limited research laboratory. Therefore, iPS cell culture is still dependent on manual techniques. Cell culture conditions, such as duration of treatment with cell detachment solution, fluid flow, and seeding cell density, are difficult to control. To preserve the pluripotency of stem cells, culture requires precise control by highly skilled operators because complicating factors cause difficulty in scaling-up the stem cell culture system [12,13]. To establish an ACCS for iPS cells in a limited space, it is necessary to standardize cell culture operations.

In this study, we describe an ACCS that enables automated iPS cell culture in a commonly used cell culture incubator. We standardized the maintenance of iPS cell culture and demonstrated long-term subculture of iPS cells using a device that automates both the positioning of seeding cells on feeder cells and their passaging.

## Results and discussion

### Automated induced pluripotent stem cell culture system

**Figure 1:**
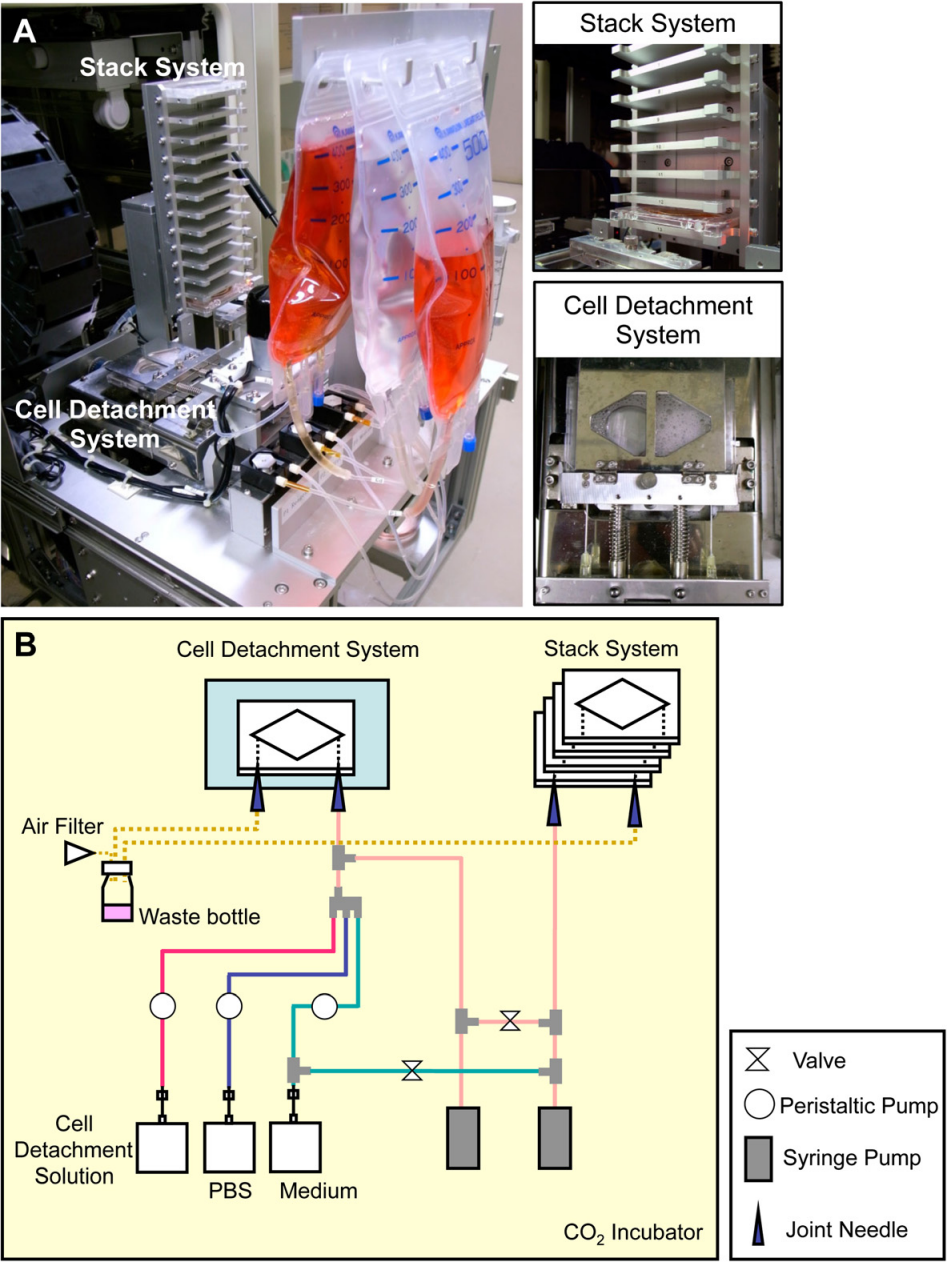
**The Automated Cell Culture System is Composed of a Cell Detachment System and Stack System.** (**A**) Photograph of automated culture system, the components of which fitted inside a commonly used CO_2_ incubator. (**B**) Schematic illustration of the whole system with components connected by closed flow path.

In the development of a mass production system for iPS cells, it is desirable that a uniform quality of cultured cells is maintained for a long-term. Stem cell culture is dependent on manual processes performed by skilled technicians at all stages [12]. Therefore, quality and safety is limited by the technique and skill of the worker [14]. In particular, iPS cells are very difficult to handle, as they have a tendency to change state easily upon each passage or operation because of which it is difficult to obtain consistent results with iPS cells. Therefore, it is necessary to automate the operations for a series of cultures. We developed a culture system capable of providing a stable supply of normal mouse iPS cells using ACCS (Figure 1A). This device automates stem cell culture, allows optimization, and enhances safety. ACSS automatically performed injection/aspiration of cell and liquid by the rotation of peristaltic pumps and the switching of the flow paths. Detachment system could dissociate the adherent cells by giving vibration to the culture chamber. Parameters such as fluid flow rate, volume, dilution ratio, enzymatic reaction time, and detachment time, were optimized and can be controlled through external PC.

ACCS was designed to be as compact as possible for industrial use (Figure 1B). It requires no centrifugation step for cell collections. As described above, iPS cells need careful maneuver; therefore, we suggest the need for an automated system for mass production in a biomedical or pharmaceutical plant. This small system is suitable for commercial adoption because it can be incorporated flexibly into a plant.

### Cell culture in a disposable cell chamber (DCC)

**Figure 2:**
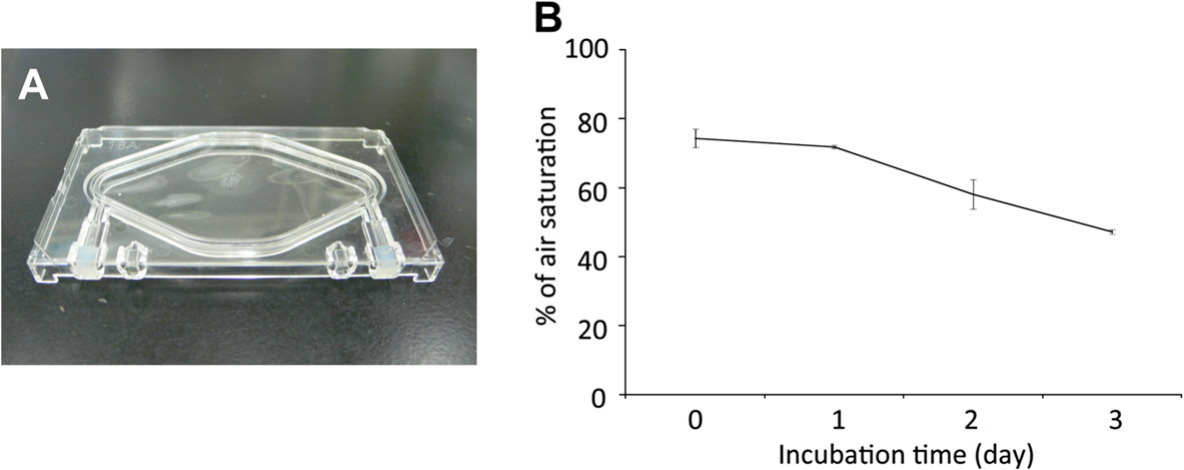
**Disposable Cell Chamber Exchanged Oxygen through Air Filter, Leading to the Proliferation of Cells.** (**A**) Photograph of DCC used in this study. (**B**) Dissolved oxygen concentration in DCC. Concentration of dissolved oxygen was measured each day for 3 days. On Day 0, iPS cells.

Mouse iPS cell cultivation was carried out in chamber uniquely designed for ACCS (Figure 2A). A disposable cell chamber (DCC) was a closed chamber with a total volume of 4.65 ml. We dispensed 1 × 10^5^ iPS cells into each DCC. Oxygen demand is a critical factor in stem cell culture; therefore, tight control of the culture environment was necessary [15]. In DCC, air exchange was performed through an aerated filter on top face. Dissolved oxygen levels in the medium in the chamber were maintained between 50–80% of the saturation of air after 2 days of cell culture (Figure 2B), comparable to conventional cell culture using a dish (data not shown).

### Passaging of induced pluripotent stem cells in the automated culture device

**Figure 3:**
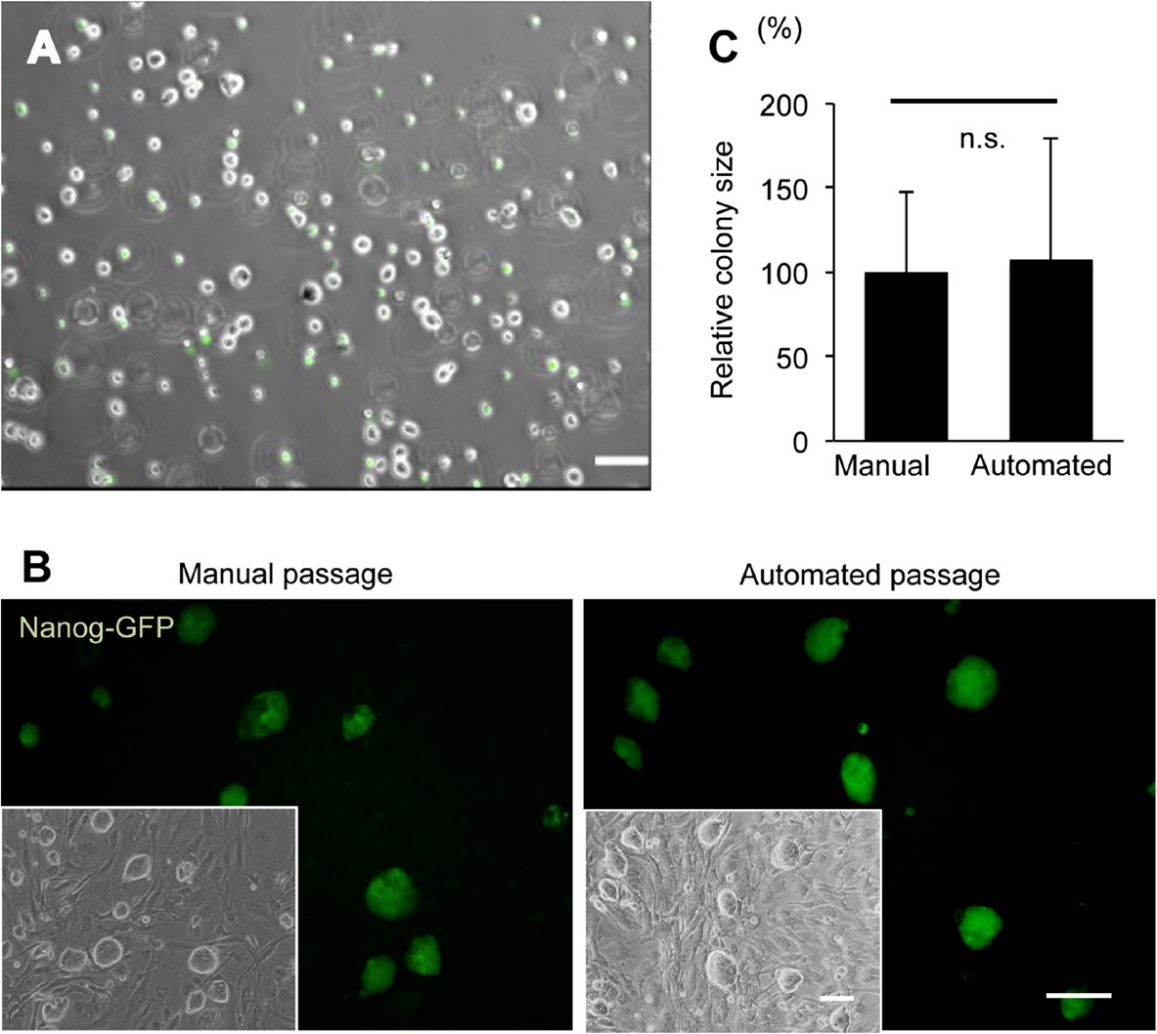
**Mouse Induced Pluripotent Stem Cells Passaging by Automated Cell Culture System.** onfluent cells were were plated on to mouse embryonic fibroblast feeder cells. Dissolved oxygen concentration was measured aseptically using an optical sensor at RT. The data is shown as mean ± standard deviation; number of chambers: 3.

In iPS cell culture, cell dissociation is the most important step. Usually, stem cell dispersion during manual passage is carried out using enzymatic and mechanical methods that are least disruptive to pluripotency [16]. iPS cells should remain as single cells from suspension to passage because a majority of the cells would not persist in a pluripotent state if they are not dissociated into single cells [17]. We optimized the method using both enzyme treatment and mechanical dissociation to achieve single cell automatic passage (Figure 3A). To monitor iPS cell pluripotency during cell culture, we utilized the fact that iPS cells are derived from transgenic mice line in which green fluorescent protein (GFP) is under the control of the Nanog promoter [18]. The Nanog-GFP transgenic mice express GFP in a pattern that is identical to that of Nanog, a known pluripotent marker.

Detached iPS cells, suspended with shaking incubation in the cell detachment unit after a 30 min-protease treatment, dissociated into single cells. After dissociation, cells were collected and plated in new DCCs through a tube with inserted needle. Morphological analysis showed that iPS cells were successfully dissociated into single cells after the passaging process (Figure 3A). No difference was observed in morphology and size of mouse iPS cell colonies between the DCC culture and manual culture in a dish (Figure 3B,C). Moreover, the iPS cell colonies in DCC showed intense Nanog-GFP fluorescence after automated culture (Figure 3B).

### Morphological analysis of induced pluripotent stem cells over multiple passages in the automated culture device

**Figure 4:**
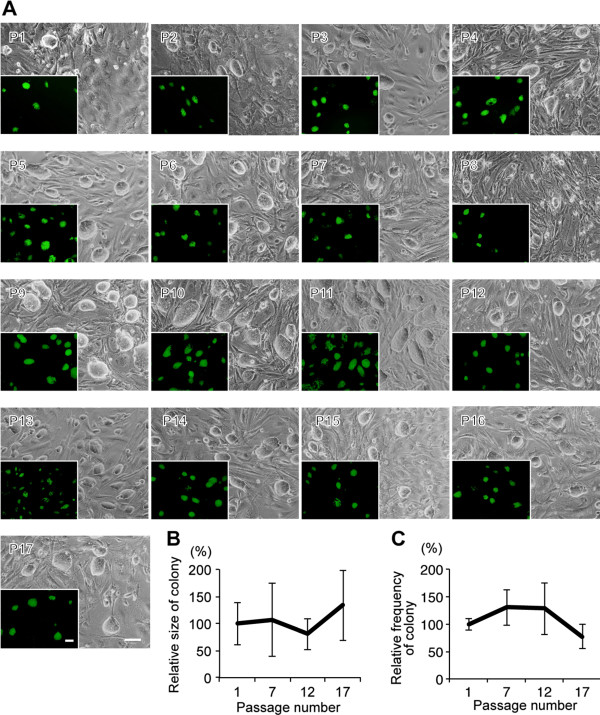
**Long-Term Induced Pluripotent Stem Cell Culture using Automated Cell Culture System.** (**A**) Morphology and corresponding fluorescence of GFP, under the control of the Nanog promoter, of each mouse iPS colony passaged automatically for long-term culture. The mouse iPS cells were cultured for 4 weeks and passaged 17 times. Scale: 100 μm. (**B**) Quantification of iPS cell proliferation based on colony size by Nanog-GFP expression compared with the first passage iPS cells. (**C**) Quantification of iPS cell viability based on colony forming frequency by Nanog-GFP expression compared with the first passage iPS cells.

The growth characteristics of iPS cells propagated in ACCS were comparable to those propagated manually. Repeated medium changes and passaging every 2 days by the automated method so that did not compromise the integrity of the iPS cell culture. No microbial contamination was observed, even after 4 weeks. Throughout the long-term culture, there were no morphological changes in mouse iPS cells cultured by the automated culture system (Figure 4A). No significant change was observed in Nanog-GFP positive areas and frequency during culture, suggesting that the undifferentiated state, growth rate and viability of iPS cells was maintained for over 17 passages, i.e., about 4 weeks of culture (Figure 4B, C).

### Expression of pluripotency-associated markers over multiple passages in automated culture device

**Figure 5:**
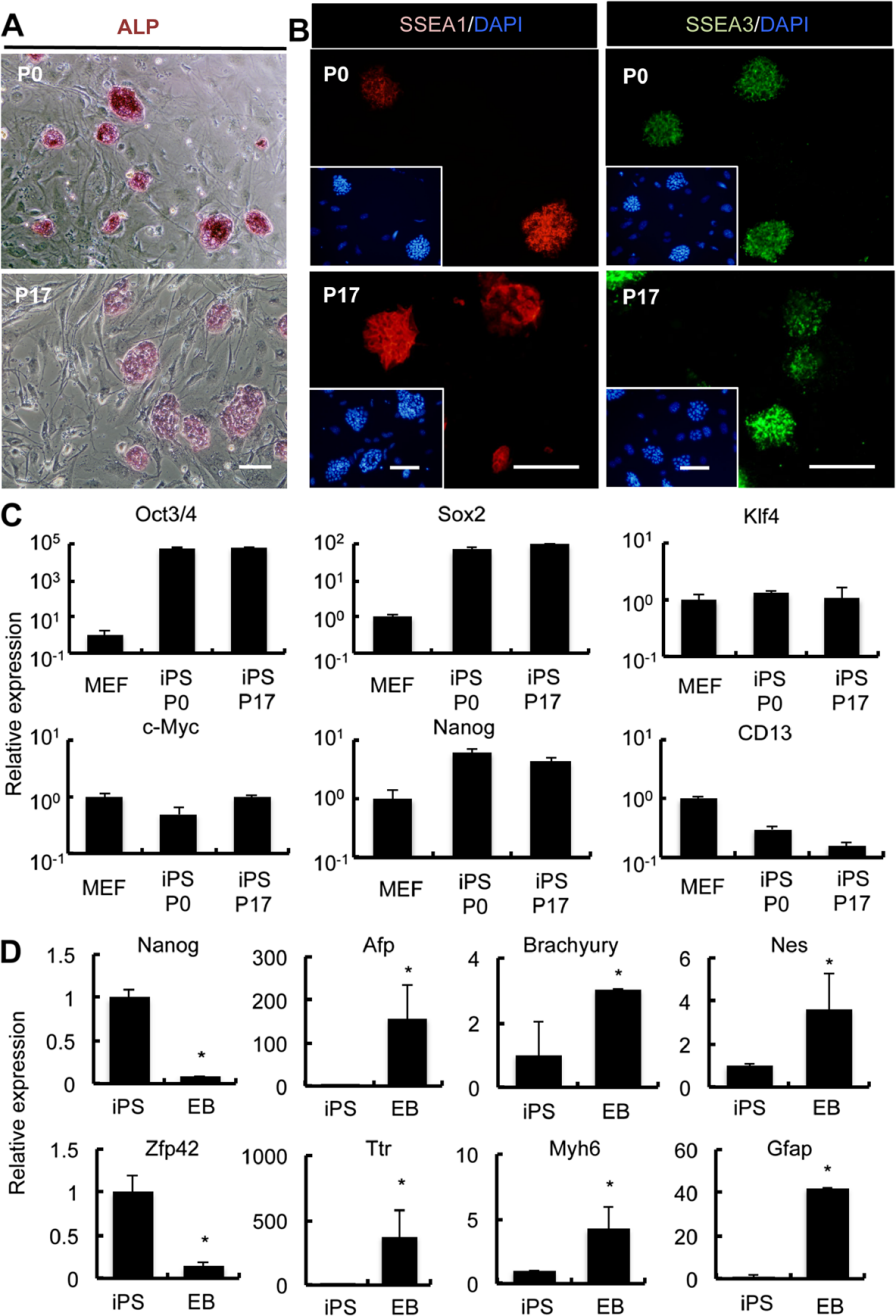
**Undifferentiated State of Induced Pluripotent Stem Cells after Long-Term Culture using Automated Cell Culture Device.** (**A**) Alkaline phosphatase activity of iPS cells at 0 and 17 passages. Scale: 100 μm. (**B**) Immunostaining of the pluripotency markers SSEA-1 and SSEA-3 in iPS cells at 0 and 17 passages. Scale: 100 μm. (**C**) Quantitative PCR analysis of the gene expression of the stem cell markers Oct3/4, Sox2, and Nanog, and the fibroblast marker CD13 compared with the MEFs. (**D**) Quantitative PCR analysis of the gene expression in EBs derived from automatically cultured iPS cells. The expressions of pluripotency markers; Zfp42, Nanog, endoderm markers; Ttr, Afp, mesoderm markers; Myh6, Brachyury, and ectoderm markers; Gfap, Nes, were compared with the iPS cells. *Statistically significant (p <0.05).

Next we examined the expression of pluripotent markers in iPS cells in automated culture over a period of 4 weeks using immunohistochemistry, alkaline phosphatase assay, and quantitative Reverse Transcription Polymerase Chain Reaction (qPCR). Stage-specific embryonic antigen (SSEA)-3, which is expressed in human embryonal carcinoma cells, is widely used for the identification of stem cells [19,20], whereas SSEA-1 is expressed in undifferentiated mouse iPS cells [21,22]. We showed that during 4 weeks of automated culture, the expression of SSEA-1 and SSEA-3 was maintained in mouse iPS cell cultures (Figure 5A). Undifferentiated iPS cells are known to exhibit tissue nonspecific alkaline phosphatase activity [22]. Using alkaline phosphatase assay we observed that the alkaline phosphatase activity was maintained for over 4 weeks (Figure 5B). Furthermore, we examined the expression of pluripotency markers by quantitative qPCR. Nanog, Oct3/4, and Sox2 mRNAs have all been identified as undifferentiated cell marker genes in iPS and embryonic stem (ES) cells [23], while CD13 is expressed in fibroblasts [24]. After 4 weeks of automated culture, expression of these marker mRNAs was equivalent to the pre-culture levels (Figure 5C). These results indicate that the automated cell culture device did not alter gene expression levels for pluripotency-associated transcription factors, indicating that maintenance culture of mouse iPS cells is possible for long-term.

To evaluate whether the iPS cells cultured with ACCS have maintained differentiation property, embryoid bodies (EB) were generated after period of culture. EB were plated on the dish and cultured for additional 2days. Expression of the differentiation markers was examined by qPCR. Gene expression of ectoderm, mesoderm, and endoderm markers were increased respectively. This result indicated that iPS cells cultured with ACCS have maintained the multi-lineage differentiation potential.

## Conclusion

In this study, we established an automated culture that enabled multiple passages of mouse iPS cells without affecting their pluripotency. We believe that ACCS established in this study has met the requirement of standardized iPS cell quality, which was verified by morphology, proliferation, and expression of markers for undifferentiated cells. In a recent study, it was demonstrated that mouse iPS cells are closer to the ground state than human pluripotent stem cells and human iPS cells can be put back in the ground state under particular culture conditions [25]. Unlike mouse iPS cells, human iPS cells must passage as cell aggregates, and dissociation into single cells leads to differentiation. It will be useful to propagate human iPS cells with ACCS by optimization of parameters, such as flow rate and disperse reaction time. It will also be possible that human iPS cells cultured with ACCS by simply applying mouse iPS cell condition establish in this study under the presence of ROCK inhibitors which enhance human iPS cell survival as a single cell [26,27]. This automated culture system, which produced a steady supply of pluripotent stem cells of constant quality, will serve as a base technology for the creation of pluripotent stem cells in bulk. For the application of these stem cells in advanced medicine, the next challenge will be to develop a method to efficiently induce differentiation of pluripotent stem cells with an automated culture system.

## Methods

### Automatic cell culture

ACCS consisted of three main components: a computer including software, control box, and cell culture system (Figure 1). The cell culture system is operated with the computer through the control box. The cell culture system is a cell-passage machine in an incubator comprising a cell detachment device, a cell chamber stack tower, and a solution exchange system. Cells that were pre-cultured in DCC were placed in the stack tower and detached mechanically in a protease solution (TrypLE Express, Life technologies Co.) after a phosphate-buffered saline wash. After detachment, the cells were dissociated by passing through a needle inserted into the DCC cell suspension diluted with culture medium, then dispensed into new DCCs in the stack tower. All steps after pre-culture were processed automatically by ACCS. Oxygen concentration in DCCs was measured using an optical oxygen sensor (Microx TX3, PreSens Precision Sensing GmbH).

### Culture of mouse induced pluripotent stem cells

The Nanog-GFP mouse iPS cells line iPS-MEF-Ng-20D-17 established as described previously [18] were cultured for 4 weeks using the automated culture device. Chambers coated with 0.1% gelatin were seeded automatically with mitomycin-inactivated mouse embryonic fibroblast (MEF) isolated from non-visceral tissues of day E13.5 mouse embryos 6 h prior to every iPS cell passaging from another chamber loaded MEF suspension. All animal experiments were approved by the Ethics Committee of Yokohama City University and were conducted according to the institutional guidelines. We plated iPS cells at a density of 10,000 cells/cm^2^. The culture medium comprised of Dulbecco’s modified Eagle’s medium (GIBCO®; Life Technologies) containing 15% Knockout™ Serum Replacement (Life technologies), GlutaMAX™ (Life technologies), non-essential amino acids, and β-mercaptoethanol. Repeated passaging was performed every 2 days by automated method. In the passage process, mouse iPS cells were dissociated with TrypLE™ Express (Life technologies) as mixture with MEFs and dispersed into single cells and diluted by fresh medium before seeding. The dissolved oxygen concentration in DCC was kept for 2 days cultivation, so that iPS cell growth would not be affected. For automated culture, centrifugation steps were performed outside the automated device. After every passage, cells were subjected to morphological evaluation. For quantification of iPS cell growth, fluorescent images were processed using IN Cell Developer Toolbox software (GE Healthcare, Fairfield, CT, USA), and the sizes of iPS cell colony were measured as GFP positive area.

### Immunocytochemical analysis

Adherent cultures were fixed with 4% paraformaldehyde for 10 min at room temperature (RT) and rinsed with phosphate-buffered saline. For SSEA-1 and SSEA-3 staining, cells were pre-incubated with 10% goat serum (Sigma-Aldrich, Missouri, USA) for 1 h at RT. Cells were incubated overnight at 4°C with the appropriate concentration of primary antibodies in 1% goat serum solution (mouse-anti-SSEA-1 1:200, Santa Cruz Biotechnology; rat-anti-SSEA-3 1:200, Millipore). Antigens were visualized using the appropriate fluorophore-conjugated secondary antibodies (Alexa Fluor**®** 555 goat anti-mouse IgG (H+L), 1:500, Life Technologies; Alexa Fluor**®** 488 goat anti-rat IgG, 1:500, Life Technologies). Nuclear staining was performed with 4′,6-diamidino-2-phenylindole (1:2,000 in Apathy’s mounting medium, Sigma-Aldrich) for 5 min. Fluorescence images were captured using a fluorescence microscope (IX-71, Olympus, Tokyo, Japan). For evaluation of cell dissociation, cells were fixed with 4% PFA for 10 min at RT and then washed with phosphate-buffered saline. Phase contrast images and fluorescence images of GFP-expressing cells were captured using a wide-field fluorescence microscope (DMI6000B, Leica Microsystems). We checked that Nanog-GFP fluorescence in iPS cells was diminished during the fixation and immunocytochemical staining operations and was negligible compared to the positive signal.

### Alkaline phosphatase assay

For alkaline phosphatase staining, cells were fixed with citrate solution with added acetone and formaldehyde for 30 s at RT, rinsed with deionized water, and treated with alkaline dye mixture (Sigma-Aldrich).

### EB formation and differentiation

For EB formation, hanging drop method was performed. Hanging drops (one droplet [30μl] contains 1000 iPS cells cultured with ACCS) were placed on the lid of a 100 mm dish filled with phosphate-buffered saline (PBS) and cultured for 7days. EBs were transferred to attachment cultures for further differentiation.

### Quantitative analysis using real-time PCR (qPCR)

After 4 weeks of automated cell culture, total RNA was isolated from mouse iPS cells using TRIzol® Reagent (Life Technologies), and reverse transcription was carried out using a High Capacity cDNA Archive Kit (Life Technologies). The PCR mix in each well included 10 μl of EagleTaq Master Mix with ROX (Roche Applied Science), 0.2 μl each of the forward and reverse primers (10 ng/μl), and 5 μl of single-strand cDNA, giving a final reaction volume of 20 μl. qPCR was performed with LightCycler® 480 system (Roche Applied Science, Germany) using the Universal ProbeLibrary (UPL) probes (Roche Applied Science). The relative quantification of gene expression was carried out according to the delta-delta Cp method. Glyceraldehyde-3-phosphate dehydrogenase (GAPDH) was chosen as the reference gene. Mouse-specific primer sequences (forward and reverse) and Universal ProbeLibrary probes are listed in Table 1. The following PCR conditions were used: 50°C for 2 min, 95°C for 10 min, 95°C for 15 s, and 60°C for 1 min, for a total of 55 cycles.

**Table 1:** qPCR Primers Used in the Present Study

**Gene**	**Forward Primer**	**Reverse Primer**	**(UPL) probe**
*Oct3/4*	GTTGGAGAAGGTGGAACCAA	CTCCTTCTGCAGGGCTTTC	#95
*Sox2*	TCCAAAAACTAATCACAACAATCG	GAAGTGCAATTGGGATGAAAA	#63
*Klf4*	CGGGAAGGGAGAAGACACT	GAGTTCCTCACGCCAACG	#62
*c-Myc*	CCTAGTGCTGCATGAGGAGA	TCTTCCTCATCTTCTTGCTCTTC	#77
*Nanog*	TTCTTGCTTACAAGGGTCTGC	AGAGGAAGGGCGAGGAGA	#110
*Zfp42*	TCTTCTCTCAATAGAGTGAGTGTGC	GCTTTCTTCTGTGTGCAGGA	#71
*Ttr*	CCTGCAGCCGCATTAAGT	GATGGTGTAGTGGCGATGG	#76
*Afp*	CATGCTGCAAAGCTGACAA	CATGCTGCAAAGCTGACAA	#63
*Myh6*	CGCATCAAGGAGCTCACC	CCTGCAGCCGCATTAAGT	#6
*Brachyury*	CAGCCCACCTACTGGCTCTA	GAGCCTGGGGTGATGGTA	#100
*Gfap*	TCGAGATCGCCACCTACAG	GTCTGTACAGGAATGGTGATGC	#67
*Nes*	TCCCTTAGTCTGGAAGTGGCTA	GGTGTCTGCAAGCGAGAGTT	#67
*CD13*	AATCTCATCCAGGGAGTGACC	TCCGCTTTAAACTGCTCCAG	#82
*Gapdh*	AGCTTGTCATCAACGGGAAG	TTTGATGTTAGTGGGGTCTCG	#9

### Statistics

Data were presented as the mean ± SD. Statistical differences were analyzed using the Mann–Whitney *U* test. P values less than 0.05 were considered significant.

## Competing interests

There are no conflicts of interest in this research.

## Authors’ contributions

HT designed the research; TT, RO, YWZ, YU and NT participated in its coordination and helped to draft the manuscript; HK, KK, and KS performed experiments and analyzed data; HK, KK, and KS wrote the paper. All authors read and approved the final manuscript.
